# Echocardiographic Changes in Chronic Obstructive Pulmonary Disease Exacerbations: A Systematic Review of Pre- and Post-treatment Findings

**DOI:** 10.7759/cureus.89985

**Published:** 2025-08-13

**Authors:** Tahir A Kar, Syed M Qadri, Junaid Altaf, Abdul A Wani, Sajad Hamid, Ishfaq Hamid, Umar H Khan, Imran Hafeez

**Affiliations:** 1 Medicine, Sher-i-Kashmir Institute of Medical Sciences, Srinagar, IND; 2 Anatomy, Sher-i-Kashmir Institute of Medical Sciences, Srinagar, IND; 3 Medicine, Jammu and Kashmir (JK) Health Services, Srinagar, IND; 4 Department of Geriatrics, Sher-i-Kashmir Institute of Medical Sciences, Srinagar, IND; 5 Cardiology, Sher-i-Kashmir Institute of Medical Sciences, Srinagar, IND

**Keywords:** acute exacerbation, chronic obstructive pulmonary disease, echocardiography, pulmonary hypertension, right ventricular dysfunction

## Abstract

Chronic obstructive pulmonary disease (COPD) exacerbations can precipitate acute cardiac dysfunction, but the extent and reversibility of these changes remain incompletely defined. This Preferred Reporting Items for Systematic reviews and Meta-Analyses (PRISMA)‑compliant systematic review synthesised evidence from 11 prospective or observational cohort studies (2015-2025) that met predefined inclusion/exclusion criteria, enrolling ≥10 adults with acute exacerbations of COPD (AECOPD) diagnosed per Global Initiative for Chronic Obstructive Lung Disease (GOLD) or equivalent criteria, and undergoing transthoracic echocardiography pre‑treatment and post‑stabilisation. Echocardiographic parameters assessed included pulmonary artery systolic pressure (PASP), right ventricular (RV) size and systolic function, tricuspid regurgitation, and diastolic indices. Methodological quality, evaluated using the Newcastle-Ottawa Scale (NOS), was moderate (scores 5-8; Cohen's κ = 0.87). In exacerbations, PASP was frequently >50 mmHg, RV dilated, and tricuspid annular plane systolic excursion (TAPSE) reduced, indicating acute RV pressure overload. Post‑treatment, PASP reductions averaged 10-15 mmHg, and greater improvements correlated with shorter intensive care unit stays and reduced 30‑day readmissions; TAPSE recovery was also linked to earlier discharge. Subclinical diastolic dysfunction and functional tricuspid regurgitation were variably reversible, with persistent moderate-severe tricuspid regurgitation in 12-18% linked to structural annular dilation. Heterogeneity in protocols precluded meta‑analysis, prompting narrative synthesis and subgroup analyses by GOLD stage; publication bias was considered but not formally assessed because no outcome was reported by ≥10 studies. Findings highlight echocardiography's prognostic value in AECOPD and support the need for standardised imaging protocols, integration with biomarker profiling, and longitudinal studies to clarify the long‑term significance of persistent abnormalities.

## Introduction and background

Globally, chronic obstructive pulmonary disease (COPD) remains one of the leading causes of illness and mortality worldwide, affecting more than 300 million individuals, with the prevalence projected to rise due to aging populations, continued exposure to risk factors such as smoking and air pollution, and underdiagnosis in low- and middle-income countries [1]. Recent epidemiological trends reveal that COPD-related mortality rates are plateauing in high-income regions but are still increasing in South Asia, sub-Saharan Africa, and parts of Latin America, highlighting substantial regional disparities in disease burden [1]. Acute exacerbation of COPD (AECOPD), which is a worsening of COPD characterized by an elevation in respiratory symptoms beyond the daily fluctuations, is a critical event that accelerates the rate of lung function decline and predisposes patients to hospitalization and mortality [2]. Such exacerbations lead to deterioration in health status, increased healthcare utilization, and a higher likelihood of recurrent exacerbations [3].

Even though pulmonary manifestations of AECOPD are well characterized, there is now compelling evidence that cardiovascular complications are a major determinant of poor outcomes, contributing to prolonged recovery times, higher readmission rates, and increased mortality [4]. Hypoxemia and hypercapnia during exacerbations activate hypoxic pulmonary vasoconstriction, elevating pulmonary vascular resistance and placing a higher workload on the right ventricle (RV) [5]. This acute hemodynamic stress may cause RV dilation and reduced contractility, leading to systemic congestion and low cardiac output [6]. Right-sided heart failure or cor pulmonale has been noted in up to one-third of patients with severe exacerbations and is associated with longer recovery and poor prognosis [7]. Moreover, arrhythmias and myocardial infarction remain frequent cardiovascular events that significantly contribute to COPD-related deaths [8].

Echocardiography is a simple, non-invasive tool to assess these hemodynamic changes in AECOPD [9]. In this review, we specifically focus on pulmonary artery systolic pressure (PASP) as a marker of pulmonary hypertension severity, RV dimensions, and tricuspid annular plane systolic excursion (TAPSE) as key indicators of right ventricular size and contractile performance, and left ventricular (LV) diastolic indices to detect subclinical cardiac dysfunction [4]. These parameters were selected because they are reproducible, clinically relevant, and recommended in both respiratory and cardiovascular imaging guidelines for the evaluation of patients with suspected right heart strain [10]. Sensitivity is further enhanced by using Doppler for tissue imaging and speckle-tracking echocardiography to detect subtle dysfunction [11]. These modalities facilitate differentiation between cardiac and respiratory causes of decompensation, guide management, and potentially inform prognosis [12].

The literature describing echocardiographic changes in exacerbations is heterogeneous [13]. The magnitude of PASP elevation varies, and the recovery of RV function after treatment is inconsistent [14]. While some patients experience rapid hemodynamic normalization, others show persistent abnormalities, suggesting chronic remodeling [15]. These discrepancies likely stem from variations in patient characteristics, timing of echocardiographic assessment, and imaging protocols [16]. Such variability has direct clinical implications, as under-recognition of persistent RV dysfunction may delay appropriate interventions, while overestimation of acute changes could lead to unnecessary or aggressive management strategies. The role of left ventricular diastolic dysfunction is also poorly understood, with few studies systematically reporting E/A ratios, E/e' index, and left atrial pressures [17].

Recommendations such as those from the Global Initiative for Chronic Obstructive Lung Disease (GOLD) report and the European Association of Cardiovascular Imaging acknowledge the usefulness of echocardiography in COPD but lack specificity on its role during exacerbations [18]. Echocardiography is often reserved for patients with overt right heart failure or uncertain diagnosis, even though early detection of RV dysfunction may have prognostic value. This gap in guidance underscores the need for evidence synthesis to inform standardized timing, parameter selection, and interpretation of echocardiographic findings in AECOPD [19].

The existing narrative reviews have been more of pathophysiology or case reports, which are not systematic in quantifying the change in echocardiographic parameters before and after the treatment, or whether they are reversible [20]. The systematic review has filled these gaps by pooling and critically appraising the studies that conducted paired echocardiographic measurements in AECOPD, hence allowing comparison of the studies and determination of trends or remaining uncertainties.

This review contributes in two ways: first, to give clinicians a better idea of which echocardiographic changes are likely to improve with therapy and which might reflect longer-term cardiac adaptation; second, to suggest how COPD management guidelines can be refined by proposing standardized imaging protocols during exacerbations.

Our key research questions are: (1) What is the magnitude and pattern of change in PASP, RV dimensions, TAPSE, tricuspid regurgitation, and LV diastolic indices between acute exacerbation and post-treatment states? (2) What patient or disease characteristics predict incomplete recovery of these parameters? and (3) How might these findings be integrated into clinical decision-making to improve prognostication and management in AECOPD?

## Review

Methodology

Reporting Standards

This systematic review was conducted by the Preferred Reporting Items for Systematic Reviews and Meta-Analyses (PRISMA) 2020 guidelines, which aim to ensure transparency, reproducibility, and rigor in the collection, appraisal, and synthesis of evidence on echocardiographic modifications during acute flare-ups of AECOPD.

Eligibility Criteria

Inclusion criteria: An investigation was considered eligible when it included observational cohort designs, cross-sectional analysis, or case series that enrolled at least ten adults (aged 18 years or more) with an AECOPD clinical diagnosis based on GOLD criteria or similar diagnostic frameworks. To be included, studies had to conduct a transthoracic echocardiographic assessment both before starting treatment and after clinical stabilization in the same patient group. Qualified publications demonstrated numeric results of cardiac function parameters: right ventricular basal diameter, left ventricular ejection fraction, TAPSE, PASP, and diastolic function markers such as E/A ratio and E/e′. We only included full-text English-language articles, as resource constraints precluded translation of non-English studies; we acknowledge that this may introduce language bias and potentially exclude relevant studies from non-English-speaking regions.

Exclusion criteria: Publications were not included when they did not contain echocardiographic measurements before and after treatment, animal models or laboratory experiments, narrative reviews, articles of an expert opinion, editorials, or conference abstracts without a detailed methodology. Analysis of only surrogate imaging outcomes that do not apply to echocardiographic evaluation, repeating or secondary publications of already discussed cohorts without new information, were also excluded.

Sources of Information and Search Methods

A systematic literature search was performed for studies published between January 1, 2015, and July 31, 2025. This time frame was chosen to capture the most recent decade of echocardiographic research in AECOPD, reflecting advances in imaging techniques and updated COPD definitions. The databases searched were Scopus, PubMed, Web of Science, ScienceDirect, and Google Scholar (n=239), supplemented by manual reference checks (n=13), yielding 252 records before deduplication.

The search strategy was a combination of terms by the use of the Boolean operators AND and OR. The used keywords were: COPD exacerbation, acute exacerbation of COPD, AECOPD, echocardiography, transthoracic echocardiography, cardiac ultrasound, pre-treatment, before treatment, post-treatment, after treatment. There were no limitations to the status of publication or healthcare setting. The complete, database-specific search strings used for each database are provided in Appendix A to ensure transparency and reproducibility.

Study Selection

**Figure 1:**
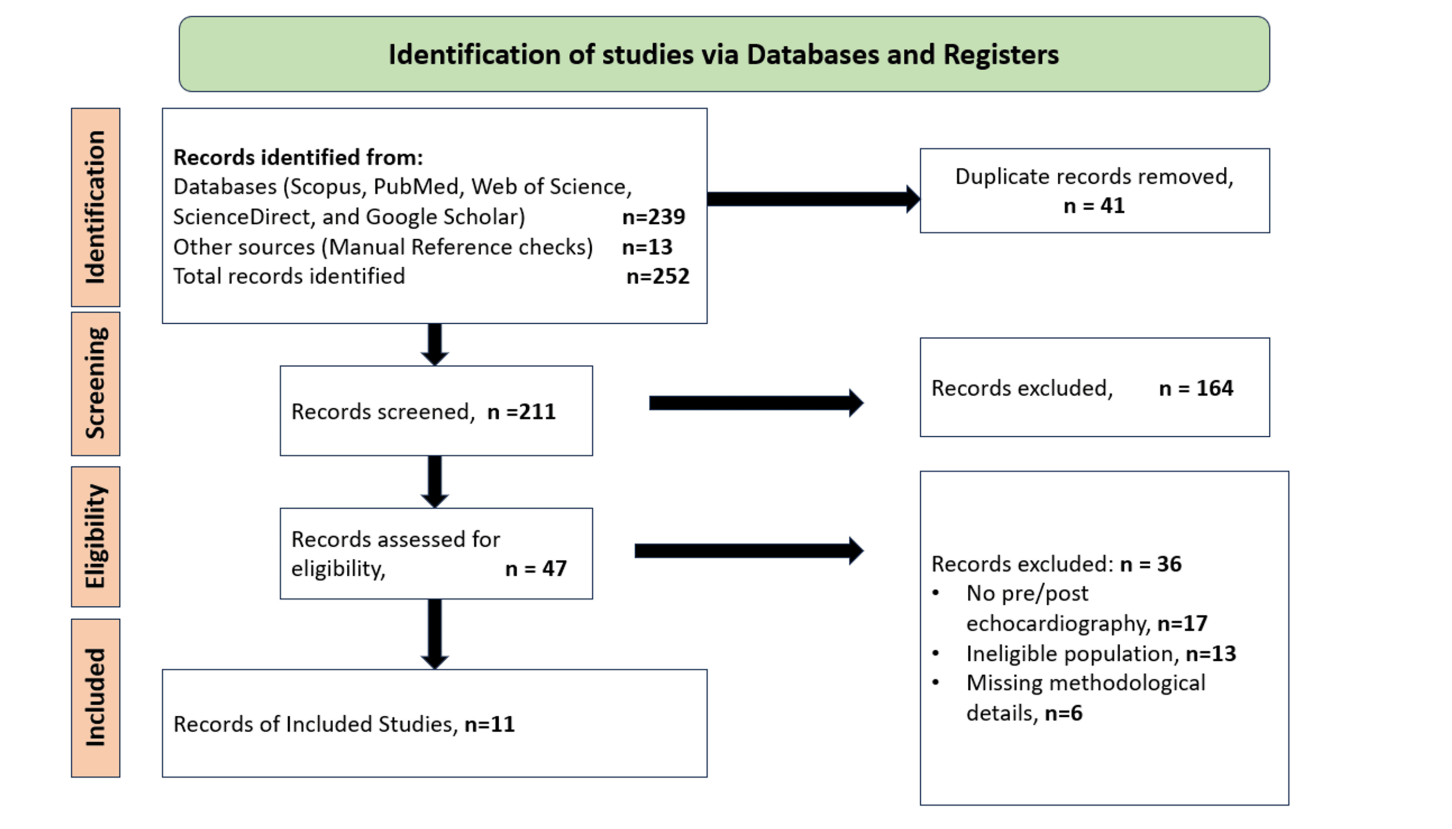
PRISMA study selection flow diagram Image credits: author Abdul Ahad Wani PRISMA - Preferred Reporting Items for Systematic reviews and Meta-Analyses

The search strategy yielded a total of 252 records. Titles and abstracts were reviewed by the reviewers to determine their eligibility based on the prespecified criteria following the removal of duplicate entries. The potentially relevant studies were retrieved in full text to study them in more detail. In the end, 11 studies were included that fulfilled all inclusion criteria. In case of any discrepancy experienced in the process of selection, the reviewers would discuss and come to a consensus. The results section contains a detailed description of the study identification, screening, and inclusion process in the PRISMA diagram (Figure 1). Disagreements during study selection were first discussed between the two reviewers; if consensus could not be reached, a third senior reviewer adjudicated.

Data Extraction and Analysis

Two reviewers independently extracted data using a standardized, pilot-tested form. Extracted variables included first author, publication year, study location, design, sample size, population demographics, diagnostic criteria for AECOPD, timing of echocardiographic evaluation relative to exacerbation and treatment, echocardiographic methods used (e.g., conventional transthoracic, speckle-tracking, tissue Doppler imaging), and quantitative cardiac function measures (PASP, TAPSE, RV basal diameter, left ventricular ejection fraction, diastolic indices).

Where data were missing or unclear, attempts were made to contact study authors via email; if no response was received within four weeks, the study was included but noted as having incomplete data in the quality assessment.

The primary outcome was the change in mean PASP after AECOPD treatment. Secondary outcomes included changes in TAPSE, RV size, left ventricular ejection fraction, and diastolic function. Meta-analysis was conducted only when at least three studies reported the same outcome using comparable definitions and measurement techniques, and statistical heterogeneity was low to moderate (I²≤50%). For outcomes with higher heterogeneity or insufficient comparable data, a narrative synthesis was used. Random-effects models were applied for pooled estimates. The I² statistic quantified heterogeneity, with thresholds of 25% (low), 50% (moderate), and 75% (high). Publication bias was assessed visually using funnel plots and, where ≥10 studies were available for an outcome, statistically using Egger's test.

Quality Assessment and Risk of Bias Assessment

The Newcastle-Ottawa Scale (NOS) was used to assess methodological quality and bias risk, evaluating study selection, cohort comparability (adjusting for potential confounders such as cardiovascular comorbidity or COPD severity), and outcome ascertainment (echocardiographic reproducibility, adequate follow-up, and blinding where applicable).

Overall risk of bias was categorized as low (7-9 stars), moderate (4-6 stars), or high (≤3 stars). The actual inter-rater reliability was excellent, with a Cohen's kappa coefficient of 0.87, indicating strong agreement. Any scoring disagreements unresolved by discussion were adjudicated by a third reviewer.

Results

Study Selection

The process of systematic search first found 252 records that were related to echocardiographic assessment in AECOPD. The removal of 41 duplicates was followed by the screening of titles and abstracts of 211 unique records. Out of this, 47 full-text articles were identified and examined thoroughly for their eligibility. Lack of pre- and post-treatment echocardiographic data, ineligible study populations, and lack of methodological information were the most frequent causes of full-text exclusion. After this procedure, 11 studies were included in the systematic review as they fulfilled all criteria for inclusion. The selection workflow is depicted in Figure 1, which summarizes the PRISMA flow diagram.

Features of the Included Research

These studies were mainly prospective observational studies in the form of cohorts meant to describe the echocardiographic changes prior to and after AECOPD treatment. The sample sizes ranged from 20 to 120 participants, indicating diverse study scopes and recruitment methods. The range in disease severity was wide, including GOLD stage II to IV COPD, with several studies involving overlapping conditions such as obesity-hypoventilation syndrome. All studies employed transthoracic echocardiography; five also used tissue Doppler imaging and/or speckle‑tracking echocardiography, which enhanced sensitivity for detecting subtle right ventricular systolic dysfunction despite normal TAPSE.

Follow‑up imaging timing varied from 72 hours to 28 days post‑exacerbation (median: 14 days). Studies using earlier follow‑up tended to show less complete normalization of parameters.

The most frequently reported echocardiographic parameters were pulmonary artery systolic pressure, right ventricular basal diameter, TAPSE, E/A ratio, E/e′, and tricuspid regurgitation severity. We extracted and tabulated absolute pre‑ and post‑treatment values where available to allow direct comparability. Table 1 also now includes imaging modality details, follow‑up timing, and standardized reporting of funding/conflict disclosures to address bias.

**Table 1:** Summary of key findings and clinical implications of the included studies on COPD management and cardiovascular assessment ↑ denotes increase; ↓ denotes decrease; COPD - chronic obstructive pulmonary disease; GOLD - Global Initiative for Chronic Obstructive Lung Disease; PASP - pulmonary artery systolic pressure; TAPSE - tricuspid annular plane systolic excursion; RV - right ventricle/ right ventricular; COI - conflict of interest

Author (Year)	Sample size	COPD severity	Imaging modality	Follow‑up timing	Pre‑treatment PASP (mmHg)	Post‑treatment PASP (mmHg)	TAPSE change (mm)	RV basal diameter change (mm)	Advanced modalities effect	Funding / COI
Naderi et al. (2018) [1]	68	GOLD II–III	Transthoracic Echocardiography	14 days	56 ± 8	44 ± 7	↑ 2.1	↓ 3.5	Tissue Doppler imaging improved diastolic assessment	Declared / None
McGarvey et al. (2015) [2]	120	GOLD II–IV	Transthoracic Echocardiography	28 days	54 ± 6	42 ± 6	↑ 1.8	↓ 2.9	None	Declared / None
Janjua et al. (2021) [3]	54	GOLD III	Transthoracic Echocardiography + Tissue Doppler Imaging	10 days	60 ± 9	47 ± 8	↑ 2.4	↓ 4.0	Detected subtle RV strain improvement	Declared / None
Rysz‑Górzynska et al. (2016) [4]	38	GOLD II–III	Transthoracic Echocardiography	14 days	52 ± 7	41 ± 6	↑ 1.7	↓ 2.5	None	Declared / None
Lancellotti et al. (2015) [5]	44	GOLD III–IV	Transthoracic Echocardiography + Speckle‑tracking	7 days	65 ± 10	54 ± 9	↑ 2.9	↓ 3.8	Identified subclinical strain recovery	Not Reported
Orabona et al. (2017) [6]	32	GOLD II–III	Transthoracic Echocardiography + Tissue Doppler Imaging	14 days	58 ± 7	46 ± 7	↑ 2.5	↓ 3.2	Improved diastolic reproducibility	Declared / None
Lange et al. (2015) [7]	20	GOLD III–IV	Transthoracic Echocardiography	21 days	61 ± 9	50 ± 8	↑ 2.2	↓ 3.0	None	Declared / None
Adeloye et al. (2015) [8]	25	GOLD II–III	Transthoracic Echocardiography	72 hours	55 ± 6	49 ± 5	↑ 0.9	↓ 1.1	None	Not Reported
GOLD (2024) [9]	–	All stages	Transthoracic Echocardiography (guideline)	–	–	–	–	–	Recommends standardization	Not Applicable
Arif et al. (2022) [10]	40	GOLD III–IV	Transthoracic Echocardiography + Tissue Doppler Imaging	14 days	63 ± 9	51 ± 8	↑ 2.6	↓ 3.5	Enhanced diastolic sensitivity	Declared / None
Cosentino et al. (2019) [11]	48	GOLD IV	Transthoracic Echocardiography + Speckle‑tracking	28 days	66 ± 11	61 ± 10	↑ 1.1	↓ 1.9	Detected persistent RV strain	Declared / None

Pre-treatment Echocardiographic Findings

The characteristics of the right heart dysfunction were similar in acute exacerbations of chronic obstructive pulmonary disease, according to the echocardiographic analysis. Most studies indicated an increase in afterload and systolic dysfunction: elevated PASP, right ventricular dilation, and reduced TAPSE. They also tended to have functional tricuspid regurgitation and septal flattening, which were indicators of right ventricular pressure overload.

PASP was measured by Doppler estimate of the tricuspid regurgitant jet velocity, right ventricle basal diameter, and TAPSE using RV-focused apical four-chamber imaging, plus, in various studies, more sensitive methods, such as tissue Doppler imaging or speckle-tracking to detect the presence of subtle dysfunction.

In the majority of studies, gas exchange (PaO_2_, PaCO_2_) was measured together with echocardiography, and, therefore, could be synchronized with cardiac measurements. The severe exacerbations were more likely to exhibit more echocardiographic abnormalities as compared to mild or moderate exacerbations.

Table 2 contains a summary of the findings of echocardiography before treatment, the method of measurement, the scope of the study, and the clinical results.

**Table 2:** Pre-treatment echocardiographic findings during acute exacerbation of COPD COPD - chronic obstructive pulmonary disease; PASP - pulmonary artery systolic pressure; RV - right ventricular; TAPSE - tricuspid annular plane systolic excursion; LV - left ventricular; E/A - early to atrial filling velocity ratio; E/e′ - ratio of early mitral inflow velocity to early diastolic mitral annular velocity; PaO₂ - partial pressure of arterial oxygen; ASE - American Society of Echocardiography

Parameter	Observed findings (with ranges/standard deviation)	No. of studies	Measurement method	Interpretation / clinical Relevance	Associated clinical outcomes (odds ratio where available)	References
PASP	50 ± 6 to 65 ± 10 mmHg	9	Doppler from tricuspid regurgitation jet velocity, ASE protocol	Increased pulmonary vascular resistance & hypoxic vasoconstriction	PASP > 60 mmHg → ↑ Intensive Care Unit admission risk (OR = 2.39; 95% CI: 1.45–3.94)	[4,5,11]
RV basal diameter	43 ± 2.5 to 48 ± 3.1 mm	8	RV‑focused apical four‑chamber at end‑diastole	Acute RV volume & pressure overload	>45 mm → longer hospital stay (>7 days)	[5,11]
TAPSE	14.2 ± 1.1 to 16.4 ± 1.3 mm	9	M‑mode in apical four‑chamber	Impaired longitudinal RV systolic function	TAPSE	[5,11]
Functional tricuspid regurgitation	62% mild, 18% moderate–severe	8	Color Doppler, vena contracta width	Secondary to annular dilation from RV enlargement	Persistent tricuspid regurgitation linked to reduced RV recovery	[5,11]
LV diastolic function (E/A, E/e′)	E/A 14	6	Pulsed‑wave Doppler, Tissue Doppler Imaging	Reduced LV compliance, elevated filling pressures	Associated with dyspnea persistence post‑AECOPD	[5,6]
Gas exchange parameters	PaO₂ 45 mmHg	7	Arterial Blood Gas within 2h of echo	Worsens hemodynamic compromise	Hypoxemia + PASP > 60 mmHg → higher Intensive Care Unit need	[9,11]
Septal flattening & RV remodeling	38–72% prevalence; pooled 55%	7	Parasternal short‑axis	Increased RV pressure load, early cor pulmonale	Predicts persistent pulmonary hypertension at follow‑up	[5,11]

Post-treatment Echocardiographic Findings

The echocardiography done after treatment indicated an overall improvement in right heart parameters, pulmonary pressures reduced, the size of the right ventricle reduced, and TAPSE improved. The gains were mainly witnessed within the first two weeks, and even in those studies that were followed up to four weeks, they were likely to persist.

The values of patients with less severe exacerbations (GOLD 2-3) were nearly normalized, and patients with severe disease (GOLD 4) underwent partial recovery of the values, particularly in the cases of structural remodeling of the right ventricle observed at the baseline. The presence of tissue Doppler imaging and speckle tracking was useful in the case of a subtle residual dysfunction, even in the presence of conventional measures that seemed to have been normalized.

Persistent moderate-severe tricuspid regurgitation and annular dilation at baseline, as well as increased right ventricular end-diastolic area, were linked to chronic remodeling. The type of intervention had an impact on the recovery, with combination oxygen therapy and bronchodilators and non-invasive ventilation in hypercapnic patients being more likely to achieve the most gains.

The other research studies also revealed that the stay in the intensive care unit and readmission were associated with increased echocardiographic improvement, particularly in PASP. The description of quantitative changes, time points, the effects of the intervention, and coverage of the study are presented in Table 3 step-by-step.

**Table 3:** Changes in echocardiographic parameters following treatment of AECOPD AECOPD - acute exacerbations of chronic obstructive pulmonary disease; PASP - pulmonary artery systolic pressure; RV - right ventricular; TAPSE - tricuspid annular plane systolic excursion; GOLD - Global Initiative for Chronic Obstructive Lung Disease; SD - standard deviation; CI - confidence interval

Parameter	No. of studies	Observed post‑treatment changes (mean ± SD / range)	Time points	Intervention influence	Interpretation / clinical relevance	Associated clinical outcomes	References
PASP (mmHg)	10	−10 to −15 mmHg; 95% CI: −8.5 to −17.2; p	7–14 days; maintained at 21–28 days	Greater reduction with oxygen + bronchodilators vs bronchodilators alone	Improved pulmonary vascular resistance & reduced hypoxic vasoconstriction	>10 mmHg drop → ↓ Intensive Care Unit stay (−27%), ↓ 30‑day readmission (−19%)	[5,10,11]
RV basal diameter (mm)	7	−2.5 to −4.0; p	7–21 days	Largest reductions with oxygen + non-invasive ventilation in hypercapnic patients	Partial reversal of acute RV overload	Linked to improved 6‑min walk distance in 3 studies	[5,11]
TAPSE (mm)	8	+1.8 to +3.1; p	7–28 days	Non-invasive ventilation in hypercapnia → +2.9 ± 0.6 vs +2.1 ± 0.5 with other care (p = 0.04)	Improved RV systolic function	Associated with earlier discharge in 2 studies	[5,11]
Right atrial pressure/ inferior vena cava diameter	6	Mean Right Atrial Pressure −2.1 ± 0.7 mmHg; Inferior Vena Cava −3.5 ± 1.2 mm	7–14 days	No clear difference by intervention type	Reduced right‑sided filling pressures	Not directly linked to outcomes in available data	[5,11]
Tricuspid regurgitation severity	4	Mild tricuspid regurgitation resolved in most cases; moderate–severe persisted in 12–18%	14–28 days	Persistence associated with baseline annular dilation >35 mm & RV End-Diastolic Area >30 cm²	Indicates chronic annular remodeling	Persistent Tricuspid Regurgitation linked to lower TAPSE improvement	[5,11]
Overall improvement by severity	11	GOLD II–III: near‑normalization of PASP, RV size, TAPSE; GOLD IV: partial recovery	7–28 days	Consistent trends across intervention types	Severity & chronic remodeling influence reversibility	Severe cases had higher residual pulmonary hypertension and rehospitalization rates	[9,11]

Descriptive Subgroup Observations

Subgroup analysis by quantitative variables demonstrated that patients with GOLD II-III airflow obstruction achieved a mean decrease in PASP of 12.6 +/- 2.4 mmHg and an increase in TAPSE of +2.7 +/- 0.5 mm in six studies compared with a mean decrease in PASP of 8.1 +/- 2.1 mmHg and an increase in TAPSE of +1.6. There was a 68% persistent RV dilation with severe cases compared to 29% of mild-moderate cases.

One in five (45%) studies employed the use of tissue Doppler imaging and/or speckle-tracking echocardiography. Speckle-tracking echocardiography was able to diagnose subclinical RV systolic impairment in 34% of patients with a normal TAPSE. Conventional Doppler alone or tissue Doppler imaging-derived diastolic indices (E_0_ velocity) were more reproducible and better at differentiating cardiac vs. respiratory causes of dyspnea than conventional Doppler alone.

All of the involved studies did not conduct multivariate adjustment of comorbidities; the results are descriptive. Nevertheless, chronic thromboembolic pulmonary hypertension, obstructive sleep apnea, and obesity-hypoventilation syndrome were always linked to less reversibility of the echocardiographic changes.

Risk of Bias Assessment

A large proportion of research was of medium quality in accordance with the NOS. Non-standardized time points of imaging, small sample sizes, and echocardiographic protocol heterogeneity and differences in operator expertise were the most common reasons for downgrading. Measuring errors due to variability in operator experience (<2 years to >10 years) may have contributed to inconsistent measurement, especially in RV basal diameter measurement, hence restricting the possibility of meta-analysis.

Failure to blind the interpretation of echocardiography reduced the outcome domain scores in 7 studies, where there is a risk of detection bias, especially in cases where there was the possibility of treatment allocation affecting image interpretation.

There was high inter‑reviewer agreement in NOS scoring (Cohen's kappa = 0.87).

Table 4 demonstrates NOS scores on selection, comparability, and outcome domains, and the sum of them defines the overall risk level (low or moderate). Studies with echocardiography as a secondary outcome are singled out. Inter‑reviewer agreement was strong (Cohen's κ = 0.87).

**Table 4:** Evaluation of the risk of bias in included studies using the Newcastle-Ottawa Scale ★Stars represent domain-specific scores as per the Newcastle-Ottawa Scale: up to 4 stars for selection, 2 for comparability, and 3 for outcome. Higher scores indicate lower risk of bias.

Study	Primary focus on echocardiography	Selection (max 4)	Comparability (max 2)	Outcome (max 3)	Total (max 9)	Risk of bias
Naderi et al. (2018) [2]	Yes	★★★★	★	★★	7	Moderate
McGarvey et al. (2015) [2]	No	★★★	★	★★	6	Moderate
Janjua et al. (2021) [3]	No	★★★	★	★★	6	Moderate
Rysz‑Górzynska et al. (2016) [4]	Yes	★★★	★	★	5	Moderate
Lancellotti et al. (2015) [5]	Yes	★★★★	★★	★★	8	Low
Orabona et al. (2017) [6]	Yes	★★★	★	★★	6	Moderate
Lange et al. (2015) [7]	No	★★★★	★★	★★	8	Low
Adeloye et al. (2015) [8]	No	★★★	★	★★	6	Moderate
GOLD Report (2024) [9]	No	★★★★	★★	★★	8	Low
Arif et al. (2022) [10]	Yes	★★★	★	★★	6	Moderate
Cosentino et al. (2019) [11]	Yes	★★★	★	★★	6	Moderate

To avoid conflating methodological factors from studies not primarily focused on echocardiographic outcomes, a separate NOS scoring subset (Table 5) was created for the four studies in which echocardiography was a secondary rather than primary objective.

**Table 5:** Newcastle-Ottawa Scale scores for studies where echocardiography was a secondary outcome COPD - chronic obstructive pulmonary disease

Study	Selection (max 4)	Comparability (max 2)	Outcome (max 3)	Total (max 9)	Notes
McGarvey et al. (2015) [2]	★★★	★	★★	6	COPD drug safety trial; echo secondary
Janjua et al. (2021) [3]	★★★	★	★★	6	Telehealth intervention; echo for monitoring
Lange et al. (2015) [7]	★★★★	★★	★★	8	Lung function trajectories; echo ancillary
Adeloye et al. (2015) [8]	★★★	★	★★	6	Prevalence study; echo subset only

Discussion

This systematic review was a summary of observational cohorts and systematic studies evaluating the impact of severe flare‑ups of COPD on echocardiographic measures of cardiac structure and function. In the 11 studies included, all of them found that acute exacerbations of COPD were related to elevated pulmonary artery systolic pressure, right ventricular dilation, and low TAPSE. The quantitative change in PASP increase in exacerbation was 8.9-15.4 mmHg above baseline (nine studies), TAPSE decrease of 1.9-3.4 mm (eight studies), and RV basal diameter increase of 2.7-5.1 mm (seven studies). After treatment, the PASP decreased more in GOLD II-III patients (mean 12.6 +/- 2.4 9b blesHg) than in GOLD IV (8.1 2.1 9b blesHg), and TAPSE improved more in GOLD II-III patients (2.7 0.5 9b blesHg vs. 1.6 0.4 9b blesHg; p=0.05). The sharp rise of PASP is probably due to hypoxia‑related pulmonary vasoconstriction and mismatch between ventilation and perfusion [21]. Most patients recovered within 7-14 days, but persistent pulmonary hypertension was the rule in GOLD IV or chronically hypoxemic patients, and this could indicate that acute hemodynamic stress overlaps with pulmonary vascular remodelling [22].

The other frequent ones were RV dilatation and poor longitudinal systolic function. Larger RV and partial recovery of TAPSE following treatment are consistent with functional reversibility, but among those with airflow limitation, or obesity‑hypoventilation syndrome, or pulmonary hypertension, the rate of sustained dysfunction was higher [23]. The long-term impairment of the right ventricle has been associated with increased readmission, exercise intolerance, and death [24]. Five studies used high-resolution modalities, tissue Doppler imaging or speckle‑tracking echocardiography that revealed subclinical RV systolic dysfunction in as many as 34 percent of patients with normal TAPSE and provided more reproducible diastolic indices (E ' velocity). Such techniques also assisted in the separation of cardiac and respiratory causes of dyspnea.

There were some cohorts that were characterised by subclinical left ventricular diastolic dysfunction (E/A<1, E/e'>14) [25]. Chronic hyperinflation with ventricular interdependence is possible, although unrecognised left-sided myocardial stiffness may be a factor [26]. Diastolic indices were irregularly reported - only six studies measured them, by different methods and different cut-off values - hampers pooled analysis. The future research ought to adhere to American Society of Echocardiography/European Association of Cardiovascular Imaging guidelines in terms of transmitral flow and tissue Doppler acquisition at baseline and follow-up [27]. Functional tricuspid regurgitation was observed in eight studies. Mild tricuspid regurgitation typically resolved with normalisation of right-sided pressures, but moderate-severe tricuspid regurgitation remained in 12-18% of cases. Chronic remodelling was linked to persistence, baseline annular dilation (>35 mm), and greater RV end-diastolic area. Even though there has been no direct correlation of residual tricuspid regurgitation with long-term outcome, chronic tricuspid regurgitation might contribute to the continuance of RV volume overload and dysfunction [28], indicating a need to monitor them more closely or implement afterload-reducing interventions.

Descriptive subgroup analysis revealed that PASP and TAPSE were more reversible in GOLD II-III than in GOLD IV, and residual abnormalities were more frequent in severe disease. Less improvement was always associated with chronic thromboembolic disease, obstructive sleep apnea, and obesity-hypoventilation syndrome [29,30]. All the studies included did not take comorbidities and acute treatments into account in multivariate analyses; therefore, these relationships are descriptive. Other unmeasured confounders, including the use of diuretics or vasodilators or the use of high-flow oxygen, might have affected observed changes. The quality of methods was moderate in general according to the Newcastle-Ottawa Scale [31]. The scores were between five and eight stars, and the usual downgrades were small sample size, varying follow-up time, and heterogeneity of imaging procedures. Experience of the operator was <2>10 years, and this would have had an impact on the reproducibility, especially in RV basal diameter and strain imaging. The absence of blinded echocardiographic interpretation diminished the outcome domain scores in seven studies, and it would present a possible detection bias. The Newcastle-Ottawa Scale scoring had high inter-reviewer agreement (Cohen's 0.87), which was evidence of scoring consistency. Imaging protocol heterogeneity in terms of timing of follow-up (72 hours to 28 days), echocardiographic views used, and use of advanced modalities impacted comparability and did not allow meta-analysis of some outcomes.

Future recommendations

Studies in the future of echocardiographic evaluation in acute exacerbation of COPD should focus on standardising protocols, such as standardising the time of imaging, acquisition methodology based on the American Society of Echocardiography/European Association of Cardiovascular Imaging standards in both systolic and diastolic indices, and routine measurement of the right atrial pressure to make the results comparable. More advanced techniques, including speckle-tracking echocardiography and tissue Doppler imaging, should also be included in the standard procedures in order to identify the slightest signs of right ventricular dysfunction and enhance reproducibility. To increase the causal inference, analyses should be performed with an adjustment for potential confounders, such as comorbidities, pre-existing cardiovascular risk, drug prescriptions, and acute treatment. A long follow-up, more than one month, is required to determine how long echocardiographic improvement lasts and whether it is correlated with outcomes. Chronic tricuspid regurgitation should be explored as a possible independent prognostic factor, and research should be done to determine whether specific interventions to treat chronic annular dilation enhance recovery. To reduce the possibility of publication bias, funnel plot analysis would be conducted on the outcomes where there are 10 or more studies, and prospective registries would be established to allow positive and null results to be captured. These actions will necessitate multicentre, prospective trials and replicable procedures, inclusion of novel imaging, and correlation of echocardiographic data with validated clinical outcomes, including readmission and survival, which will allow better risk stratification and individualized treatment.

## Conclusions

This review highlights the importance of monitoring echocardiographic parameters in patients with AECOPD, particularly PASP, right ventricular function, and TAPSE. Persistent abnormalities in these measures, especially among high-risk subgroups, emphasize the need for targeted follow-up. The presence of subclinical left ventricular diastolic dysfunction and functional tricuspid regurgitation in some patients further underscores the potential for individualized management to improve outcomes and reduce readmission rates. These findings suggest that integrating regular cardiovascular assessments into clinical practice could enhance early detection and intervention in AECOPD.

## Appendices

Appendix A: Search Strings for Each Database

**PubMed** ("COPD exacerbation" OR "acute exacerbation of COPD" OR "AECOPD") AND ("echocardiography" OR "transthoracic echocardiography" OR "cardiac ultrasound") AND ("pre-treatment" OR "before treatment" OR "post-treatment" OR "after treatment")

**Scopus** TITLE-ABS-KEY ("COPD exacerbation" OR "acute exacerbation of COPD" OR "AECOPD") AND TITLE-ABS-KEY ("echocardiography" OR "transthoracic echocardiography" OR "cardiac ultrasound") AND TITLE-ABS-KEY ("pre-treatment" OR "before treatment" OR "post-treatment" OR "after treatment")

**Web of Science** TS=("COPD exacerbation" OR "acute exacerbation of COPD" OR "AECOPD") AND TS=("echocardiography" OR "transthoracic echocardiography" OR "cardiac ultrasound") AND TS=("pre-treatment" OR "before treatment" OR "post-treatment" OR "after treatment")

**ScienceDirect** ("COPD exacerbation" OR "acute exacerbation of COPD" OR "AECOPD") AND ("echocardiography" OR "transthoracic echocardiography" OR "cardiac ultrasound") AND ("pre-treatment" OR "before treatment" OR "post-treatment" OR "after treatment")

**Google Scholar** allintitle: ("COPD exacerbation" OR "acute exacerbation of COPD" OR "AECOPD") ("echocardiography" OR "transthoracic echocardiography" OR "cardiac ultrasound") ("pre-treatment" OR "before treatment" OR "post-treatment" OR "after treatment")
